# Analysis of influencing factors of sleep quality in severe trauma patients without continuous sedation

**DOI:** 10.3389/fmed.2025.1707861

**Published:** 2026-01-05

**Authors:** Yue Jin, Shuya Wang

**Affiliations:** Department of Trauma Center, The Second People’s Hospital of Changzhou, The Third Affiliated Hospital of Nanjing Medical University, Changzhou, Jiangsu, China

**Keywords:** sleep quality, severe trauma, influencing factors, without continuous sedation, trauma

## Abstract

**Aim:**

Sleep disorders are very common and serious among trauma patients, particularly in those with severe trauma, and have a negative impact on patients’ physiological functioning. This study aimed to explore the status and influencing factors of sleep quality in severe trauma patients receiving non-continuous sedation.

**Methods:**

The prospective observational study was conducted in our hospital. A total of 139 patients receiving non-continuous sedation for severe trauma were selected as the research objectives. Objective: Sleep quality was continuously monitored for seven consecutive nights using the Fitbit Charge 2 to measure total sleep time (TST), while subjective sleep quality was assessed each morning using the Richards–Campbell Sleep Questionnaire (RCSQ). Demographical, clinical, environmental, and psychosocial variables were collected concurrently. Univariate and multiple linear regression analyses were performed to identify the predictors of sleep quality at different time points.

**Results:**

The average nighttime TST among the participants was 299.76 ± 85.22 min, and the mean RCSQ score was 51.38 ± 13.67, indicating generally poor sleep quality. Both objective and subjective sleep measures were poorest on day 1 and showed gradual improvement through day 7. Multivariate regression analyses identified several major predictors of reduced sleep quality, including pain intensity, thirst severity, environmental noise and light levels, ward temperature, pre-admission sleep status, trauma site (limbs), anxiety level, duration of visits, use of sleep aids, and the frequency of night-time non-invasive procedures.

**Conclusion:**

Severe trauma patients without continuous sedation experience markedly impaired sleep during the early post-injury period, with gradual improvement over time but persistent effects from multiple clinical, environmental, and psychological factors. The findings highlight several modifiable determinants—such as pain control, environmental management, and reduced nighttime procedures—that may serve as key targets for clinical sleep-promoting interventions in trauma care.

## 1 Introduction

Trauma refers to the injuries caused by mechanical factors to the human body, resulting in damage to tissue structures or impaired function (1). With the acceleration of urbanization in China, the incidence of various traumatic events has been increasing year by year. Among these, the mortality and disability rates of patients with severe trauma remain high, imposing a significant burden on both patients and their families (2).

Sleep disorders refer to various functional disturbances in the sleep–wake process, including abnormal sleep duration, atypical behaviors during sleep, and disrupted sleep–wake rhythms (3). These disorders are common and serious among trauma patients, particularly in those with severe injuries and are manifested as insomnia, lethargy, sleep deprivation, frequent awakenings, and early-morning awakenings (4). A retrospective analysis of 1,129 orthopedic patients with acute trauma found that the incidence of sleep disorders was 51.4%, with 15.4% of patients experiencing severe sleep disturbances (5). Similarly, a study evaluating subjective sleep quality in patients with traumatic spinal cord injury found an incidence of sleep disorders as high as 74.7%, with paraplegic patients being particularly affected. Their symptoms primarily included sleep-disordered breathing, circadian rhythm disturbances, insomnia, and periodic limb movements during sleep (6). Furthermore, numerous studies have confirmed that patients with severe trauma continue to experience sleep disorders even after discharge or transfer from the intensive care unit (ICU) (7).

Sleep disorders adversely affect multiple physical functions, including reduced immunity, memory impairment, delayed wound healing, decreased exercise capacity, insulin resistance, increased pain perception, and even higher mortality (8). These disorders are particularly harmful to patients with critical trauma (9). Traumatic pain is a common cause of wakefulness, and it can trigger the production and release of various inflammatory mediators, among which IL-1 plays a key role in regulating sleep (10). A study by Gulam et al. (11) on the sleep quality of orthopedic trauma patients found that half of the participants reported pain as a major factor disturbing their sleep. Another study reported that 64.7% of orthopedic trauma patients were unable to sleep due to pain, 41.0% experienced sleep disruption caused by pain, and 27.6% woke up early because of pain (12), results that align with Gulam’s findings. Therefore, effective prevention and management of pain in patients with acute severe trauma are particularly important, and the appropriate use of analgesics and sedatives can help reduce the pain burden and improve sleep quality.

Craniocerebral injuries accounts for a large proportion of patients with severe trauma, and sleep disorders are common among these patients (13). They frequently experience insomnia, daytime sleepiness, and fragmented sleep patterns. Sleep disruption can lead to neurocognitive and behavioral deficits and may prolong the recovery period after injury (14). Therefore, early identification and management of insomnia caused by the injury itself can significantly improve the prognosis of patients with traumatic brain injury.

Currently, research on the sleep quality of trauma patients remain limited. Most studies focus on the sleep patterns of patients with severe injuries at specific sites, or on the sleep conditions of severe trauma patients following ICU transfer or hospital discharge (15). No studies have described or analyzed the sleep conditions of severe trauma patients during the early stage of injury. Therefore, this study aimed to assess the sleep quality of severe trauma patients in the early stage of injury, as well as its influencing factors, providing a reference for the scientific management of sleep quality in future clinical practice.

Unlike previous studies that focused on specific injury types, on post-ICU or post-discharge sleep disturbances, or single-time-point assessments, this study is the first to continuously evaluate both objective and subjective sleep quality during the early acute stage (days 1–7) in severe trauma patients *without continuous sedation*. This approach enables characterization of short-term sleep trajectories and identification of early-phase factors uniquely influencing sleep quality.

## 2 Data and methods

### 2.1 General data

A total of 139 patients with severe trauma admitted to a Grade-III general hospital between January and December 2022 were enrolled using a convenience sampling method. Inclusion criteria were as follows: (1) age ≥18 years; (2) Injury Severity Score (ISS) > 16 (40); (3) no continuous use of sedative drugs within 24 h, with a Richmond Agitation-Sedation Scale (RASS) score > − 3; (4) clear consciousness, Glasgow Coma Scale (GCS) score >14 (41), and ability to cooperate with the sleep scale assessment; and (5) provision of informed consent.

Exclusion criteria included: (1) severe traumatic brain injury with impaired consciousness; (2) history of major depression, schizophrenia, or other psychiatric disorders; (3) cognitive or communication impairments due to Alzheimer’s disease, intracranial space-occupying lesions, or other causes.

Shedding criteria included: (1) patients who could not be observed for full 7 days during the investigation due to various reasons (such as those who developed impaired consciousness due to disease progression, those who required renewed sedation for more than 24 h, or those discharged before 7 days); and (2) patients unable to complete full-night sleep monitoring due to discomfort from wearing the health tracker. This study was approved by the Ethics Committee of The Second People’s Hospital of Changzhou, the Third Affiliated Hospital of Nanjing Medical University (Approval No: CZ2201_L007; Date: January 2022). Written informed consent was obtained from all the participants or their legal representatives prior to enrollment.

### 2.2 Methods

#### 2.2.1 Objective sleep status survey (total sleep time monitoring at night)

In this study, the Fitbit Charge 2 (Fitbit, San Francisco, United States), a smart health-monitoring device, was used to dynamically record patients’ night-time sleep duration as an objective indicator of sleep quality. The device is designed to provide improved feedback on different sleep stages by combining activity data and heart rate variability. When the body enters sleep, limb movement and muscle tone decrease; therefore, sleep–wake patterns can be indirectly reflected by monitoring periods of rest and movement of the extremities. Additionally, as the heart beats, capillaries expand and contract in response to changes in blood volume. The Fitbit Charge 2 uses an optical heart rate sensor that emits green light-emitting diode (LED) light multiple times per second, while a photodiode detects volume changes in the capillaries above the wrist to determine real-time heart-rate measurements. By continuously monitoring heart-rate variability throughout sleep, the device estimates the duration of each sleep stage—including light sleep, deep sleep, and rapid eye movement (REM) sleep. Thus, by wearing Fitbit Charge 2, sleep-related metrics—such as total sleep time, time awake after sleep onset, light sleep time, deep sleep time, and sleep efficiency—can be derieved through analysis of nighttime activity and heart-rate patterns. Some international studies have suggested that the use of Fitbit devices may offer several health benefits to patients, helping to maintain their motivation for rehabilitation exercise (16). However, another study reported that when Fitbit Charge 2 was applied to non-intubated ICU patients, only total sleep time (TST) could be reliably and continuously recorded, whereas data on awakenings (AW) and detailed sleep stages could not be fully captured. Therefore, in this study, the objective sleep assessment using Fitbit Charge 2 focused primarily on collecting patients’ nighttime total sleep duration.

During implementation, a fully charged Fitbit Charge 2 was secured around the patient’s wrist. To optimize signal quality and comfort, the device was preferentially placed on the non-injured upper limb. For patients with injuries to both upper limbs or no upper limb involvement, the non-dominant arm was selected. Moreover, the device was always positioned on the arm opposite to the one used for routine non-invasive blood pressure monitoring to avoid interference from cuff inflations.

#### 2.2.2 Subjective sleep status survey

Subjective sleep status was evaluated using the Chinese version of the Richards–Campbell Sleep Questionnaire (RCSQ) (17), which was completed independently by each participants. The translated scale has demonstrated a reliability coefficient of 0.889 and a validity of 0.84, confirming its suitability for assessing sleep quality in critically ill patients in China. The RCSQ includes five items: sleep latency, sleep depth, frequency of awakenings, ease of returning to sleep, and overall sleep quality. Each item is scored on a 0–100 mm visual analog scale (with 1 cm equivalent to 10 points). A score out of 100 is assigned for each item based on the previous night’s sleep. The total RCSQ score is calculated as the mean of the five item scores. A total score > 75 indicates good sleep quality, 25–75 indicates poor sleep quality, and < 25 indicates very poor sleep quality, with lower scores reflecting worse sleep quality.

During the sleep monitoring period, trained researchers visited patients each morning between 08:00 and 09:00. Using a standardized script, they explained the purpose and content of the survey and provided clear instructions on how to complete the questionnaire. Patients then filled out the scale independently. If a patient was unable to complete the form on their own, the researchers asked the patient about their sleep experience from the previous night (20:00 to 08:00) and recorded the responses orally provided by the patient on the RCSQ. All questionnaires were collected on site. Invalid responses—such as incomplete answers or those showing obvious patterned responses—were excluded from the analysis.

#### 2.2.3 Data completeness

The completeness of objective sleep data was rigorously monitored throughout the study. Across the seven-day monitoring period for all 139 patients—representing a total of 973 potential patient-nights of recording—valid TST data were successfully obtained for 926 nights, resulting in a data completeness rate of 95.2%. The 47 missing nights of data resulted from the following operational factors, unrelated to patient discomfort: temporary device removal during scheduled surgeries or medical procedures (*n* = 38), device charging (*n* = 7), and occasional failure of the device to synchronize data (*n* = 2). This high rate of successful data acquisition supports the robustness of the objective sleep analysis.

### 2.3 Statistical analysis method

Statistical analyses were conducted using SPSS 26.0 (IBM, Armonk, New York, United States). Normally distributed continuous data were expressed as mean ± standard deviation and compared between two groups using independent samples t-tests; comparisons among three or more groups were conducted using one-way analysis of variance (ANOVA). Non-normally distributed continuous data were summarized as median (P25, P75) and compared between the two groups using the Wilcoxon rank-sum test, while the Kruskal–Wallis test was applied for comparisons across three or more groups. Before conducting multiple linear regression analyses, multicollinearity among independent variables was assessed using variance inflation factors (VIF). A VIF > 5 was considered indicative of significant multicollinearity; however, no variables exceeded this threshold, and all were retained in the final models. Potential confounders were addressed by including clinically relevant variables, as well as those with *p* < 0.05 in univariate analyses. The Pearson correlation coefficients were used to assess the relationship between two normally distributed continuous variables, whereas the Spearman correlation coefficients were applied for non-normally distributed data. Data collected at different time points were analyzed using repeated-measures ANOVA with Greenhouse–Geisser correction where applicable. Post-hoc comparisons between time points and groups were performed using the Bonferroni method. A two-sided *p*-value < 0.05 was considered statistically significant.

## 3 Results

### 3.1 Subject recruitment results

**Figure 1:**
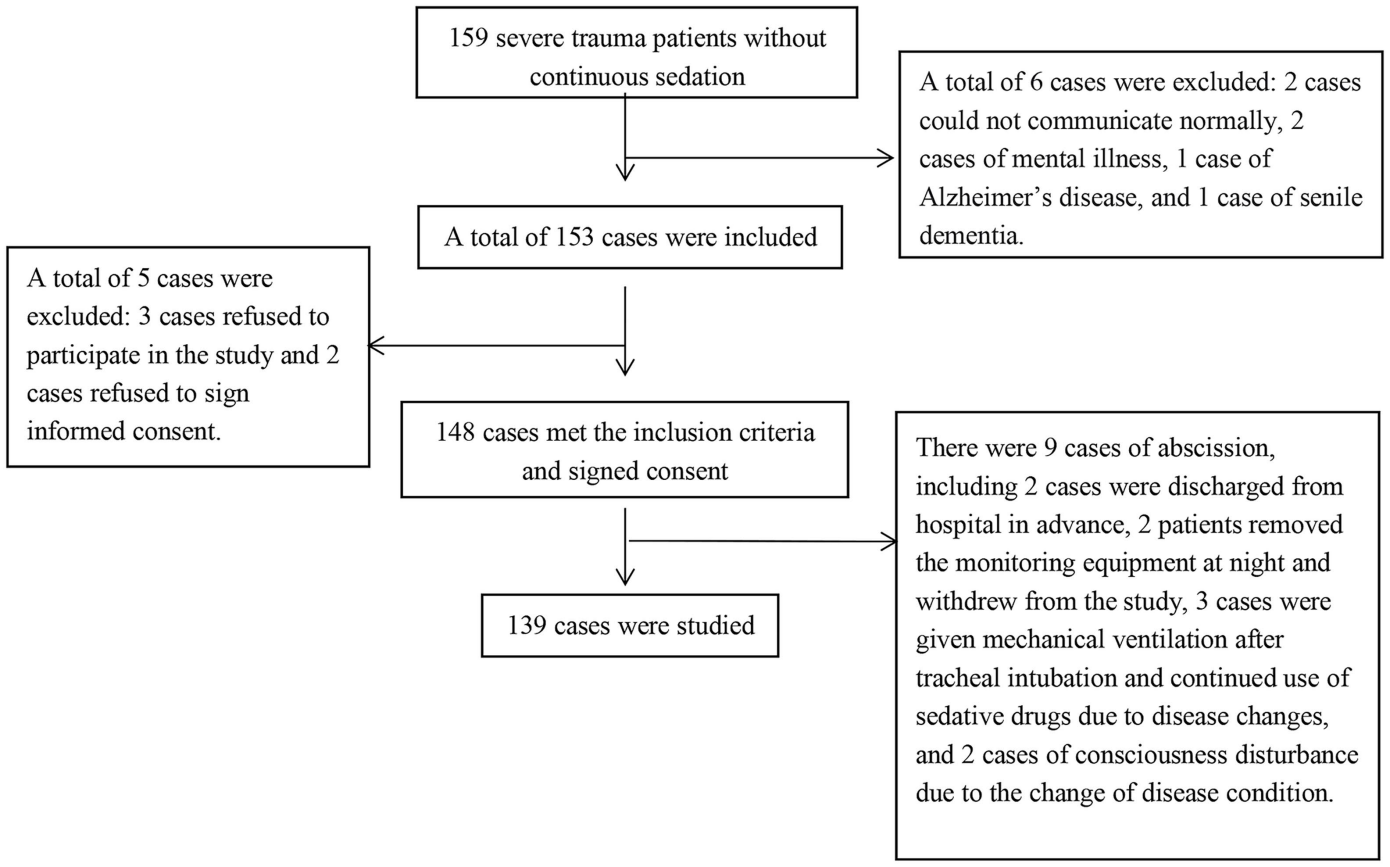
Subject recruitment flow chart.

A total of 159 patients with severe trauma were initially recruited, of whom 148 met the inclusion criteria and were enrolled. During the study period, nine participants were excluded according to the shedding criteria, resulting in 139 patients who completed the full investigation. The detailed recruitment process is shown in Figure 1.

### 3.2 General demographic information of the study subjects

General demographic data collected included age, sex, education level, marital status, employment status, payment method, history of sleep problems, chronic diseases, and injury history. The study included 109 males and 30 females, with a mean age of 49.93 ± 15.14 years. In terms of educational background, 98 patients had completed middle school or below, and 41 had completed high school or above. Employment status was as follows: 89 employed, 8 unemployed, 29 retired, and 13 not employed. In terms of payment methods, 41 were self-pay, 43 were covered by medical insurance, and 55 were covered by commercial insurance. Regarding marital status, 21 patients were married but living without their spouse present, while 118 were married with spouse. Prior sleep problems were reported by 12 patients, whereas 127 reported no such history. Additionally, 51 patients had a history of chronic disease and 88 did not; 22 reported a previous injury history, while 117 had none. Detailed general characteristics of severe trauma patients are presented in Table 1.

**Table 1:** General data of severe trauma patients (*n* = 139).

Items	*n* (%)/(^−^x ± s)
Gender	
Male	109 (78.42)
Female	30 (21.58)
Average age (years)	49.93 ± 15.14
Level of education	
Secondary school and below	98 (70.50)
High school and above	41 (29.50)
Marital status	
No spouse (single, divorced, widowed)	21 (15.11)
Have a spouse	118 (84.89)
Employment status	
Never employed	13 (9.35)
Unemployment	8 (5.76)
Retired people	29 (20.86)
On job	89 (64.03)
Payment method	
Self-paying	41 (29.50)
Medical insurance	43 (30.93)
Commercial insurance	55 (39.57)
Sleep before admission	
No	127 (91.37)
Yes	12 (8.63)
Chronic diseases	
No	88 (63.31)
Yes	51 (36.69)
History of injury	
No	117 (84.17)
Yes	22 (15.83)

### 3.3 Overall sleep quality

#### 3.3.1 Objective sleep quality

The mean total sleep time at night was 299.76 ± 85.22 min. The majority of patients slept less than 5 h per night.

#### 3.3.2 Subjective sleep quality

The total RCSQ score was 51.38 ± 13.67, indicating a moderate-low level of subjective sleep quality. Scores for each dimension were as follows: sleep depth 47.94 ± 16.32, sleep latency 51.26 ± 19.97, frequency of awakenings 48.76 ± 17.07, ease of returning to sleep 53.03 ± 18.67, and overall sleep quality 55.93 ± 16.60. Detailed results are shown in Table 2.

**Table 2:** Total score of the RCSQ scale and the scores of each dimension of the study subjects (*n* = 139).

Items	*x- ± s*
Sleep depth	47.94 ± 16.32
Sleep time	51.26 ± 19.97
Wake times	48.76 ± 17.07
Return to sleep	53.03 ± 18.67
Sleep quality	55.93 ± 16.60
Total RCSQ score	51.38 ± 13.67

RCSQ, Richards–Campbell sleep questionnaire.

#### 3.3.3 Correlation analysis of subjective and objective sleep quality

Pearson correlation analysis was used to assess the relationship between objective and subjective sleep measures in non-sedated severe trauma patients. Results showed that night-time sleep duration on days 1, 3, 5, and 7 was positively correlated with RCSQ sleep quality scores (*p* < 0.001), indicating a good consistency between the two assessment methods (Table 3).

**Table 3:** Correlation between subjective and objective sleep quality.

Indicators	Sleep duration on day 1		Sleep duration on day 3		Sleep duration on day 5		Sleep duration on day 7	
	*r*	*P*	*r*	*P*	*r*	*P*	*r*	*P*
RCSQ score on day 1	0.772	<0.001						
RCSQ score on day 3			0.640	<0.001				
RCSQ score on day 5					0.576	<0.001		
RCSQ score on day 7							0.606	<0.001

RCSQ, Richards–Campbell sleep questionnaire.

#### 3.3.4 Comparison of sleep quality at different time points

##### 3.3.4.1 The quality of sleep varies at different time points

Subjective and objective sleep quality were compared at different time points following enrollment. Significant differences were observed in both TST and RCSQ scores across days 1, 3, 5, and 7 (*p* < 0.001). Post-hoc analysis of objective sleep quality indicated significant differences between the following pairs: day 1 and day 3, day 1 and day 5, day 1 and day 7, day 3 and day 5, and day 3 and day 7 (*p* < 0.001). No significant difference was found between day 5 and day 7 (*p* > 0.05). For subjective sleep quality, significant differences were observed between day 1 and day 3, day 1 and day 5, day 1 and day 7 (*p* < 0.001), as well as between day 3 and day 5 and day 3 and day 7 (*p* < 0.05). No statistically significant difference was found between day 5 and day 7 (*p* > 0.05). Results are detailed in Tables 4, 5.

**Table 4:** Status of sleep quality of subjects at different time points after enrollment (*n* = 139).

Items	Day 1 of enrollment	Day 3 of enrollment	Day 5 of enrollment	Day 7 of enrollment	*F*	*p*
	(^−^*x ± s*)	(^−^*x ± s*)	(^−^*x ± s*)	(^−^*x ± s*)		
Total sleep duration	246.30 ± 100.90	296.74 ± 82.51	327.29 ± 69.49	328.71 ± 53.79	33.47	<0.001
RCSQ score	43.49 ± 14.35	51.69 ± 13.31	55.09 ± 12.57	55.25 ± 10.90	25.56	<0.001

RCSQ, Richards–Campbell sleep questionnaire.

**Table 5:** Comparison of sleep quality between groups at different time points after the enrollment of study subjects.

Time Point Comparison	Total sleep duration		RCSQ score	
	*t*	*p*	*t*	*p*
T1 vs. T2	−5.77	<0.001	−6.807	<0.001
T1 vs. T3	−8.785	<0.001	−9.046	<0.001
T1 vs. T4	−8.762	<0.001	−8.7	<0.001
T2 vs. T3	−3.627	<0.001	−2.932	<0.05
T2 vs. T4	−3.786	<0.001	−2.721	<0.05
T3 vs. T4	−0.223	>0.05	−0.181	>0.05

T1 indicated the first day of enrollment; T2 represented the third day of enrollment; T3 indicated the 5th day of enrollment. T4 indicated the 7th day of group entry. RCSQ, Richards–Campbell sleep questionnaire.

##### 3.3.4.2 Trends in sleep quality over time

**Figure 2:**
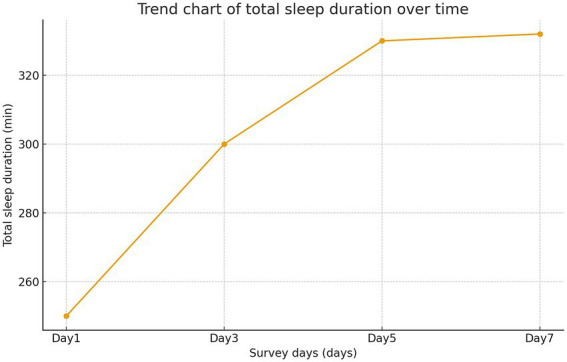
Trend of total sleep time (TST) over time.

**Figure 3:**
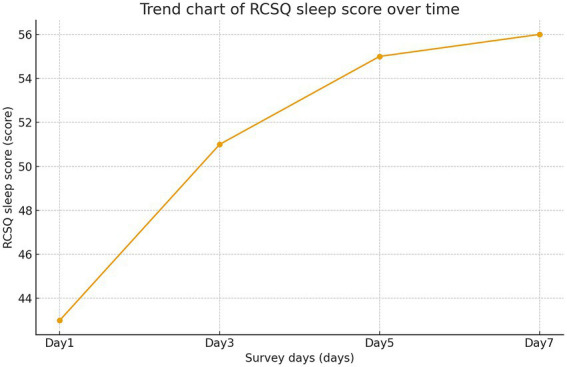
Trend chart of RCSQ sleep score over time. RCSQ, Richards–Campbell sleep questionnaire.

On day 1, mean TST was 246.30 ± 100.90 min and mean RCSQ score was 43.49 ± 14.35. By day 3, TST increased to 296.74 ± 82.52 min and RCSQ score to 51.69 ± 13.31. On day 5, TST was 327.29 ± 69.49 min and RCSQ score was 55.09 ± 12.57. By day 7, TST reached 328.71 ± 53.79 min and RCSQ score was 55.25 ± 10.90. Monitoring over seven consecutive days indicated that sleep quality remained generally poor, with the lowest levels observed on the first day. However, both TST and RCSQ scores showed a consistent increasing trend over time, as shown in Figures 2, 3.

### 3.4 Influencing factors of sleep quality

According to the inclusion criteria, the study monitored the sleep quality of the subjects for 7 days after enrollment and investigated the influencing factors, focusing on the analysis of the influencing factors of sleep quality on the 1^st^, 3^rd^, 5^th^, and 7^th^ days after enrollment. With TST and RCSQ score as dependent variables and sleep quality as independent variables, univariate analysis was performed. The items with significant differences in the univariate analysis were taken as independent variables, and the TST RCSQ score was taken as dependent variable to complete the meta-linear regression analysis. The assignment method of argument variables was shown in Supplementary Table 1.

#### 3.4.1 Factors influencing sleep quality on the first day of enrollment

##### 3.4.1.1 Single factor analysis of influencing factors of sleep quality

###### 3.4.1.1.1 Objective sleep quality

Factors associated with TST included trauma site (extremities), gastrointestinal symptoms, respiratory disturbance or dyspnea, VAS dyspnea score, cough, nocturnal cough symptom score, thirst severity score, pain score, muscle strength, mean noise level, mean light intensity, minimum ward temperature, maximum ward temperature, mean ward temperature, ward type, availability of recreational facilities in the room, hospital anxiety scale score, hospital depression scale score, duration of visits, use of sleep aids, and invasive and non-invasive procedures.

###### 3.4.1.1.2 Subjective sleep quality

Factors associated with RCSQ score included gastrointestinal symptoms, VAS dyspnea score, cough, nocturnal cough symptom score, thirst severity score, pain score, mean noise level, mean light intensity, minimum ward temperature, maximum ward temperature, mean ward temperature, ward type, availability of recreational facilities, hospital anxiety scale score, hospital depression scale score, duration of visits, use of sleep aids, and invasive and non-invasive procedures (*p* < 0.05 and *p* < 0.01; see Supplementary Table 2).

##### 3.4.1.2 Multiple linear regression analysis of influencing factors of sleep quality

###### 3.4.1.2.1 Objective sleep quality

Multiple regression analysis indicated that TST was associated with thirst severity, pain score, mean light intensity, use of sleep aids, and non-invasive procedures (*p* < 0.05; Supplementary Table 3).

###### 3.4.1.2.2 Subjective sleep quality

RCSQ score was associated with pain and mean light intensity (*p* < 0.05; Supplementary Table 4).

#### 3.4.2 Factors influencing sleep quality on the third day of enrollment

##### 3.4.2.1 Single factor analysis of influencing factors of sleep quality

###### 3.4.2.1.1 Objective sleep quality

Factors associated with TST included trauma site (abdomen), body temperature, gastrointestinal symptoms, nocturnal cough symptom score, thirst severity score, pain score, physical restraint status, maximum noise level, mean noise level, mean light intensity, minimum ward temperature, maximum ward temperature, mean ward humidity, ward type, availability of recreational facilities, hospital anxiety scale score, hospital depression scale score, type of daytime visits, duration of visits, use of sleep aids, and non-invasive procedures.

###### 3.4.2.1.2 Subjective sleep quality

Factors associated with RCSQ score included body temperature, gastrointestinal symptoms, cough, nocturnal cough symptom score, thirst severity score, pain score, maximum noise level, mean noise level, mean light intensity, maximum ward temperature, mean ward humidity, ward type, availability of recreational facilities, hospital anxiety scale score, hospital depression scale score, duration of visits, use of sleep aids, and invasive procedures (*p* < 0.05 and *p* < 0.01; Supplementary Table 5).

##### 3.4.2.2 Multiple linear regression analysis of influencing factors of sleep quality

###### 3.4.2.2.1 Objective sleep quality

Multiple regression analysis showed that TST was associated with thirst severity score, pain score, mean noise level, mean light intensity, and use of sleep aids (*p* < 0.05; Supplementary Table 6).

###### 3.4.2.2.2 Subjective sleep quality

RCSQ score was associated with maximum noise level and mean noise level (*p* < 0.05; Supplementary Table 7).

#### 3.4.3 Factors influencing sleep quality on the 5th day of enrollment

##### 3.4.3.1 Single-factor analysis of influencing factors of sleep quality

###### 3.4.3.1.1 Objective sleep quality

Factors associated with TST included payment method, absence of injury history, ISS score, trauma site (extremities), body temperature, gastrointestinal symptoms, type of oxygen therapy, respiratory disturbance or dyspnea, VAS dyspnea score, nocturnal cough symptom score, thirst severity score, pain score, mean noise level, mean light intensity, mean ward temperature, hospital depression scale score, type of daytime visits, duration of visits, use of sleep aids, and invasive and non-invasive procedures.

###### 3.4.3.1.2 Subjective sleep quality

Factors associated with RCSQ score included payment method, pre-admission sleep status, ISS score, trauma site (extremities), dyspnea, VAS dyspnea score, thirst severity score, pain score, mean noise level, mean light intensity, type of daytime visits, duration of visits, and use of sleep aids (*p* < 0.05 and *p* < 0.01; Supplementary Table 8).

##### 3.4.3.2 Multiple linear regression analysis of influencing factors of sleep quality

###### 3.4.3.2.1 Objective sleep quality

Multiple regression analysis indicated that TST was associated with payment method, ISS score, trauma site (extremities), mean noise level, mean light intensity, mean ward temperature, and use of sleep aids (*p* < 0.05; Supplementary Table 9).

###### 3.4.3.2.2 Subjective sleep quality

RCSQ score was associated with pre-admission sleep status, trauma site (extremities), mean noise level, and use of sleep aids (*p* < 0.05; Supplementary Table 10).

#### 3.4.4 Factors influencing sleep quality on the 7th day of enrollment

##### 3.4.4.1 Single factor analysis of influencing factors of sleep quality

###### 3.4.4.1.1 Objective sleep quality

Factors associated with objective sleep quality (total sleep duration) included payment method, presence or absence of chronic disease, ISS score, trauma site (extremities), gastrointestinal symptoms, pain score, mean noise, hospital anxiety scale score, visiting duration, and use of sleep aids.

###### 3.4.4.1.2 Subjective sleep quality

Factors associated with patients’ subjective sleep quality (RCSQ score) included payment method, sleep status before admission, presence or absence of chronic disease, site of trauma (extremities), gastrointestinal symptoms, pain score, mean noise, minimum light intensity, hospital anxiety scale score, visitation duration, and use of sleep aids (*p* < 0.05 and *p* < 0.01; Supplementary Table 11).

##### 3.4.4.2 Multiple linear regression analysis of influencing factors of sleep quality

###### 3.4.4.2.1 Objective sleep quality

Multiple regression analysis showed that total sleep duration was correlated with pain score, mean noise, hospital anxiety scale score and use of sleep aids (*p* < 0.05, Supplementary Table 12).

###### 3.4.4.2.2 Subjective sleep quality

Multiple regression analysis showed that RCSQ score was correlated with sleep condition before admission, trauma site (limbs), mean noise, visiting duration, and use of sleep aids (*p* < 0.05, Supplementary Table 13).

## 4 Discussion

Sleep disorders are very common and serious in trauma patients, especially in severe patients, which seriously affect patients’ health (9). Our results demonstrated that the total sleep duration (objective sleep quality) of severe trauma patients at night is less than 5 h, which was similar to the results of a study by foreign scholar Elliott (18), while the sleep time of normal adults should be 7–9 h (19). The patient’s RCSQ score (subjective sleep quality) and scores in all dimensions were in the lower middle level, which was consistent with the research results (20). The above results show that patients with severe trauma have less total sleep time at night, low sleep efficiency, difficulty falling asleep during ICU hospitalization, easy to wake up frequently under the influence of the external environment, shallow overall sleep, and not easy to enter deep sleep. Studies have confirmed that efficient and sufficient sleep can promote the brain to enter deep sleep, which can not only maintain the energy of patients, but also improve the body’s immunity, which is of great help to the physical rehabilitation of patients (21). However, the subjects of this study were all patients with severe trauma, mainly injured from car accidents, falling from high places and heavy objects. Such patients have serious injuries, often accompanied by severe pain, and are prone to sleep disorders under the stimulation and influence of multiple factors, which are bound to have an adverse impact on the rehabilitation of their diseases. Beyond confirming previously reported influences such as pain, noise, and environmental light, our study provides additional insights by simultaneously examining both objective and subjective sleep measures in the acute post-injury period. To our knowledge, this is the first study to continuously monitor total sleep time across the first 7 days of hospitalization in severe trauma patients without continuous sedation, allowing a more comprehensive characterization of early sleep disturbances.

In the process of continuous monitoring, this study found that although the sleep quality of severe trauma patients was poor, their total sleep duration and sleep quality scores also improved with the increase in enrollment time, and the objective and subjective sleep quality gradually improved with the extension of hospitalization days. This may occur because acute trauma excites the sympathetic nervous system, increasing catecholamines. Elevated levels of norepinephrine, in particular, can promote wakefulness (22). At the same time, after suffering sudden changes, patients faced the double blow of physical and psychological, coupled with the influence of a number of factors such as the unfamiliar environment after hospitalization, the sleep of severe trauma patients was more likely to be disturbed in the early stage, and the sleep quality was worse than other time periods. With the increase of enrollment time, patients gradually had a certain adaptability to the environment, and the disease was alleviated. The influence of some factors on sleep quality gradually decreased, and sleep quality improved. This finding is consistent with previous reports that rotator cuff injuries produce nighttime pain and sleep quality disturbances that improve with treatment (23). Importantly, the temporal pattern observed in our cohort—markedly impaired sleep on day 1 followed by gradual improvement by day 3 and stabilization after day 5—has not been previously described in trauma populations. Earlier studies largely assessed sleep at a single time point or after ICU discharge, whereas our longitudinal design provides new evidence on the dynamic trajectory of sleep recovery during the acute phase.

On the fifth day of enrollment, total sleep duration at night was affected by how medical expenses were paid. Among the subjects of this study, the number of self-paying patients accounted for 29.50%, the number of medical insurance payment was 30.93%, and the number of commercial insurance payment was 39.57%, which accounted for the highest proportion. However, in this population, the self-paid patients had worse sleep quality than those paid for medicare and commercial insurance. Some domestic studies have also confirmed that medical expenses are a risk factor for low sleep quality (24).

Multiple linear regression analysis identified that sleep problems existing before admission were the influencing factors of sleep quality. In other words, patients who experienced poor sleep prior to hospitalization were more likely to have worse sleep quality after hospitalization, which was consistent with the research results of Bihari’s (25) team. The reasons were considered as follows: The original sleep disorders were further aggravated due to the influence of diseases and environmental factors, and at the same time, due to the need for fasting, gastrointestinal decompression and other measures after injury, some drugs used to promote sleep could not be taken temporarily, resulting in worse sleep quality.

ISS scoring tool is the gold standard for assessing the severity of injuries in patients, which is closely related to the site of injury and the distribution of the causes of injury, and can comprehensively reflect the injury of various anatomical sites. The higher the injury severity score (ISS), the more serious the injury of patients is (26). This study found that the ISS of patients was negatively correlated with sleep quality, meaning that the more severe the injury degree of patients, the worse the sleep quality at night. Consistently, in a previous study, it was found that the ISS of trauma patients was positively correlated with physical anxiety, and a higher degree of anxiety would seriously affect the sleep quality of patients and lead to sleep disorders (27).

In this study, it was found that the quality of sleep was different depending on the site of trauma. The sleep quality of patients with fractures of limbs was worse than that of patients without injury. In recent years, for patients with severe trauma combined with limb injury, “one-stage surgery” is generally adopted in clinical practice, and polymer plaster external fixation, stent external fixation, bone traction and other methods can minimize the degree of secondary injury in patients with limb fracture (28). However, due to the limitations of external fixation, traction, and special position, they are often in forced lying position, and the comfort of patients is significantly reduced, thus affecting the quality of sleep. Therefore, for patients with severe trauma combined with limb fractures, medical personnel should pay more attention to sleep.

In the multiple regression analysis of influencing factors on day 1 and day 3, it was found that thirst was an important factor affecting the sleep quality of patients. It has been also noted in Arai et al. (29) that severe patients usually experience severe thirst. Combined with the actual situation of the subjects in this study, the reasons for the early thirst of patients are considered as follows: When patients with severe trauma are combined with shock, they show severe thirst at first due to the loss of a large amount of blood and body fluids. In addition, some trauma patients need to undergo emergency surgery. Due to fasting and water prohibition before surgery, loss of intraoperative fluid, and inability to drink water immediately after surgery, patients are thirsty after surgery. Therefore, this study showed that the thirst of patients was more obvious in the early stage of injury, which led to the sleep disorder of patients. In addition to the above reasons, the body continues to be in a state of hypermetabolism in the earlier stage of severe trauma, and stress hyperglycemia occurs, and when the blood glucose concentration increases, the osmotic pressure of extracellular fluid increases, stimulating thirst in patients with thirst centers (30). Different degrees of thirst can lead to corresponding changes in patients’ comfort, thus leading to a decline in the quality of sleep (31). Several days after injury, patients received oral feeding or enteral nutrition as prescribed by their doctors, and the body fluid balance was restored, leading to relief of thirst symptoms and a reduced impact on sleep quality. The results of this study suggest that medical staff should strengthen the assessment of thirst in severe trauma patients in the early stage, and actively take diversified measures to correct thirst, relieve the symptoms of thirst, and further let patients get better sleep. Furthermore, our analysis identified several additional predictors that have not been systematically evaluated in previous trauma-related sleep studies—including payment method, sleep status before admission, ward temperature, and the frequency of non-invasive nighttime procedures. These findings highlight the multidimensional nature of early sleep disruption and suggest new targets for intervention.

The results of the study on days 1, 3, and 7 showed that pain was one of the main factors affecting the sleep quality of the patients, indicating that the higher the pain level of the patients, the worse the sleep quality, and for the trauma patients, the impact of pain on sleep quality persisted. Due to the large number of trauma sites and serious injuries, patients with severe trauma are often accompanied by different degrees of pain, and the duration is longer. Pain is related to wakefulness. Even though patients may have fallen asleep, pain may still disrupt their normal sleep patterns; conversely, sleep disorders may aggravate existing pain (32). Therefore, reducing pain is conducive to improving the sleep quality of patients.

The results of this study showed that noise was an influential factor in patients’ sleep quality, and on the 3rd, 5th and 7th day of monitoring, it was suggested that noise had an impact on patients’ nighttime sleep duration and subjective sleep score, indicating that noise was a persistent influencing factor. This was consistent with the findings of Martinez et al. (33) In addition to noise, another important factor affecting patients’ sleep quality is light intensity. This study found that on the first night of monitoring, light intensity had an impact on both subjective and objective sleep quality. Previous studies have shown that light intensity can affect an individual’s sleep quality and physiological parameters (34). In the early stage of severe trauma patients, the condition is serious and the treatment is complicated. To accurately and timely detect the changes in the patient’s condition and do a good job in rescue, the ward needs to always keep the light environment, which leads to patients being in a bright environment for a long time and unable to sleep. Likewise, Hilde M Wesselius et al. have indicated that the most reported sleep-disturbing factors in hospitalized patients are noise of other patients, medical devices, pain, and toilet visits (35).

The results of the study on day 5 and day 7 also found that the maximum value and average value of ambient temperature, which were two monitoring indicators, had an impact on both subjective and objective sleep of patients with severe trauma. Although the 24-h body temperature monitoring is implemented in the care ward, the perceived temperature of the patient is often ignored in this process, and the care ward is difficult to meet the special needs of individual patients for ambient temperature, if the needs of the patient are not met in a timely manner, it will lead to a decline in comfort, thereby affecting the quality of sleep of the patient.

The results of this study showed that anxiety was a psychological factor that affects sleep quality, and the higher the anxiety level, the worse the sleep quality. Many studies have confirmed the correlation between anxiety and sleep quality (36), and similar results were also obtained in this study. Patients with severe trauma often experience adverse psychological reactions to sudden injury, such as irritability, anxiety, and depression. These reactions can include mentally re-living the event, having nightmares, and worrying about their prognosis, functional recovery, and financial costs. Such psychological distress inevitably impairs sleep quality. The inclusion of both psychological and physiological variables allowed us to identify complex interactions influencing the quality of sleep, further distinguishing our study from previous work that typically focused on only one or two domains.

The usage of sleep aids was associated with sleep quality. During the investigation, it was found that when some patients were concerned about poor sleep at night, medical staff would take the initiative to intervene with drugs, such as temporary intravenous administration of midazolam and diazepam, oral administration of benzodiazepines such as sulazepam to help patients fall asleep, or oral administration of melatonin drugs such as agomelatine and rametylamine to improve sleep. This study showed that patients who used sleep aids had significantly better sleep quality than those who did not, which was consistent with previous studies (37).

This study found that the more frequent non-invasive medical procedures were performed on patients at night, the worse the subjective and objective sleep quality of patients, the more significant the impact was, and the impact was greatest in the early stage of ICU admission after injury. Previous studies have found that about 7% of patients in ICU are awakened by night nursing activities, and another 18% are awakened by nursery–patient interaction (38). A foreign study also showed that after the ICU medical staff reduced the frequency of night-time related medical activities to a minimum, the impact on the sleep quality of patients was still obvious (39). How to implement nursing work more reasonably and safely and reduce the interference on patients’ sleep still needs further exploration and research.

Furthermore, the clinical implications of these findings deserve emphasis. The strong association between pain and impaired sleep indicates that optimizing pain management—through individualized analgesic plans, multimodal analgesia, and timely reassessment—may directly improve night-time rest and accelerate recovery. Likewise, noise and light were persistent contributors to sleep disruption, suggesting that implementing structured “quiet hours,” reducing non-essential alarms, dimming lighting during night shifts, and offering earplugs or eye masks when appropriate could create a more restorative environment. Our results also show that frequent non-invasive procedures significantly interrupt sleep, particularly during the early phase after trauma. This highlights the importance of clustering nighttime nursing activities whenever clinically feasible, thereby reducing unnecessary awakenings. Meanwhile, addressing modifiable comfort factors such as thirst and room temperature may further enhance sleep continuity. By translating these modifiable risk factors into targeted interventions, healthcare providers can better integrate sleep promotion into routine trauma care and support early physiological recovery.

Overall, compared with earlier studies, our findings extend current knowledge by providing a multidimensional, time-resolved evaluation of sleep quality and its drivers during the acute phase of severe trauma. However, our study has some limitations. First, the sample size is relatively small, and the research time is short. In addition, the study used a convenience sample drawn from a single tertiary trauma center, which may limit the external validity of our findings. This sampling approach may introduce selection bias, as patients included in this hospital-based cohort may not fully represent severe trauma populations in other institutions, geographic regions, or health system contexts. Therefore, the generalizability of our results should be interpreted with caution. Second, although we placed Fitbit on the non-injured arm (or the arm opposite the blood pressure cuff) to minimize impact, we cannot fully rule out that injuries or medical procedures affecting the whole body (e.g., systemic pain, sedation) could have influenced the activity and heart rate signals, potentially affecting TST estimation. The strong correlation we observed between subjective and objective sleep measures is reassuring, but future studies with polysomnography are warranted for validation. Further large-scale and multicenter studies should be carried out to validate our findings in the near future. Another important limitation concerns the reliability of sleep measurement using the Fitbit Charge 2. As noted earlier, consumer-grade wearable devices have restricted accuracy in ICU and trauma settings, especially in distinguishing wakefulness, REM sleep, and sleep stages. Several studies have shown that Fitbit devices tend to overestimate total sleep time and underestimate wake periods when compared with medical-grade actigraphy or polysomnography (PSG). Therefore, our objective sleep data should be interpreted with caution, as the device may not fully capture fragmented sleep patterns that are common in severely injured patients. Despite these limitations, Fitbit was selected for several practical reasons. First, PSG and standardized actigraphy are difficult to implement repeatedly during the early post-injury phase because they require specialized equipment, trained technicians, and may interfere with medical treatment, patient positioning, or surgical wounds. Second, many trauma patients experience pain, restricted mobility, or the presence of medical devices, making PSG leads or actigraphy attachments uncomfortable or unsafe. In contrast, Fitbit allows continuous, non-invasive, low-burden monitoring without disrupting nursing procedures, and its high patient tolerance enables multi-day data collection that would otherwise be challenging with traditional tools. Although not a substitute for PSG, Fitbit provided a feasible and pragmatic solution for capturing longitudinal trends in this acute clinical context.

## 5 Conclusion

The sleep quality of patients with severe trauma without continuous sedation is generally poor, and the sleep quality in the early stage of injury is worse than that in other periods, and the sleep quality also improves with time. The main factors affecting the sleep quality of patients with severe trauma included payment method, sleep condition before admission, trauma site (limbs), ISS, ward noise, light intensity, ward temperature, thirst severity, pain, anxiety, duration of visits, non-invasive medical procedures, and use of sleep aids.

## Data Availability

The original contributions presented in the study are included in the article/Supplementary material, further inquiries can be directed to the corresponding author/s.
