# Supplementary material for: Analysis of influencing factors of sleep quality in severe trauma patients without continuous sedation
Source: Front Med (Lausanne). 2026 Jan 5;12:1707861. doi: 10.3389/fmed.2025.1707861 (PMC12813116; doi:10.3389/fmed.2025.1707861)
Supplement: Supplementary file 1 [file Data_Sheet_1.docx]

Supplementary Table 1 Assignment mode of independent variable

| Influencing factors | Assignment mode |
| --- | --- |
| Gender | Male=1; Female=2 |
| Level of education | Secondary school and below =1; High school and above =2 |
| Marital status | Have no spouse=1, Have spouse=2 |
| Working condition | Never employed=1, Unemployment =2; Retired people=3; On job =4 |
| Payment method | Self-paying=1; Medical insurance=2; [Commercial insurance](javascript:;) =3 |
| Sleep status before admission | No sleep problems=0; Have sleep problem=1 |
| Have chronic diseases | No=0; Yes=1 |
| Have history of injury | No=0; Yes=1 |
| ISS score | >16 scores=1; >25=2 |
| Trauma type | Traffic injury=1; Fall injury from height=2; Fall injury=3；  Injury caused by heavy objects=4; Others (machine damage, and knife wounds)=5 |
| **In-room entertainment facilities** |  |
| Head  Thorax  Belly  Pelvis  Spine  Limbs | No damage=0; Damage=1  No damage=0; Damage=1  No damage=0; Damage=1  No damage=0; Damage=1  No damage=0; Damage=1  No damage=0; Damage=1 |
| Operation situation | No operation=0; Have surgery=1 |
| Method of anesthesia  Body temperature | Unanesthesia=0; General anesthesia=1; Intraspinal anesthesia=2; Local anesthesia=3  Normal=1; Abnormal=2 |
| Gastrointestinal symptoms | None=0; Nausea=1; Hunger=2; Bloating=3; Diarrhea=4 |
| Oxygen therapy type | Artificial airway mechanical ventilation=1; High flow oxygen therapy=2; Mask oxygen=3; Double nasal oxygen=4 |
| Disturbance in respiration (or dyspnea) | No=0; Yes=1 |
| Cough | No=0; Yes=1 |
| Myodynamia  Constraint state  Room type  Entertainment facilities are provided  Visitation form  Visiting duration | Normal=1; Abnormal=2  Unconstrained=0; Constrained=1  Single room=1; Multiple rooms=2  No=0; Yes=1  No visits=0; Visitation=1  0 min=0; Within 30 minutes=1; More than 30 minutes=2 |
| Vasoactive agent | Unused=0; Used=1 |
| No analgesic drugs (including oral drugs) | Used=0; Dezocine=1; Remifentanil=2; Muscular or oral painkillers=3 |
| Sleep aid (including oral medicine) | Unused=0; Used=1 |

Note: ISS: injury severity score.

Supplementary Table 2 Correlation analysis of influencing factors of sleep quality on day 1 (*r* value)

| Items | Total sleep duration | RCSQ score |
| --- | --- | --- |
| Age | -0.053 | 0.054 |
| Gender | 0.073 | 0.068 |
| Level of education | -0.077 | -0.118 |
| Marital status | -0.005 | -0.016 |
| Working condition | -0.032 | -0.055 |
| Payment method | 0.031 | 0.057 |
| Sleep status before admission | 0.016 | -0.034 |
| Have chronic diseases | -0.054 | -0.009 |
| Have history of injury | -0.117 | -0.067 |
| APACHE-II score | 0.143 | 0.139 |
| ISS score | -0.078 | -0.103 |
| Trauma type | -0.037 | -0.044 |
| Head | 0.116 | 0.087 |
| Thorax | 0.088 | 0.076 |
| Belly | 0.094 | 0.006 |
| Pelvis | 0.054 | 0.140 |
| Limbs | -0.196^*^ | -0.066 |
| Spine | -0.027 | 0.054 |
| No operation | -0.043 | -0.041 |
| Anesthesia method | 0.012 | -0.033 |
| Body temperature | -0.095 | -0.079 |
| Gastrointestinal symptom | -0.366^**^ | -0.286^**^ |
| Oxygen therapy type | 0.159 | 0.106 |
| Disturbance in respiration (or dyspnea) | -0.193^*^ | -0.161 |
| VAS score (dyspnea score) | -0.283^**^ | -0.212^*^ |
| Cough | -0.257^**^ | -0.210^*^ |
| Nocturnal cough symptom score | -0.362^**^ | -0.319^**^ |
| Thirst severity score | -0.652^**^ | -0.487^**^ |
| Pain score | -0.705^**^ | -0.627^**^ |
| Self-care Score (Barthel) | 0.123 | 0.103 |
| Myodynamia | -0.172^*^ | -0.019 |
| Restrained condition | 0.071 | 0.084 |
| Catheter slip score | 0.015 | -0.034 |
| Minimum noise value | -0.031 | -0.069 |
| Maximum noise value | 0.005 | -0.052 |
| Mean noise value | -0.668^**^ | -0.555^**^ |
| Minimum ward illumination | 0.107 | 0.127 |
| Maximum ward illumination | 0.098 | 0.087 |
| Mean light intensity | -0.818^**^ | -0.700^**^ |
| Minimum ward temperature | -0.309^**^ | -0.316^**^ |
| Maximum ward temperature | -0.299^**^ | -0.318^**^ |
| Mean ward temperature | -0.273^**^ | -0.294^**^ |
| Minimum ward humidity | 0.026 | 0.044 |
| Maximum ward humidity | 0.110 | 0.026 |
| Mean ward humidity | 0.053 | 0.015 |
| Ward type | -0.266^**^ | -0.228^**^ |
| Whether entertainment facilities are available in the room | -0.218^**^ | -0.182^*^ |
| Hospital anxiety scale score | -0.473^**^ | -0.327^**^ |
| Hospital depression scale score | -0.504^**^ | -0.381^**^ |
| Day visiting form | 0.136 | 0.058 |
| Visiting duration | 0.340^**^ | 0.238^**^ |
| Use of vasoactive drugs | 0.138 | 0.135 |
| Pain medication use | -0.077 | -0.076 |
| Use of sleep AIDS | 0.384^**^ | 0.316^**^ |
| Invasive operation | -0.238^**^ | -0.220^**^ |
| Noninvasive operation | -0.390^**^ | -0.297^**^ |

Note: ^*^*P*<0.05, ^**^*P*<0.01. RCSQ: Richards-Campbell sleep questionnaire; APACHE-II: Acute Physiology and Chronic Health Evaluation II; ISS: injury severity score; VAS: visual analogue scale.

Supplementary Table 3 Multiple regression analysis of factors influencing objective sleep quality on day 1

|  | Regression coefficient | Standard error | Standardized regression coefficien | *t* value | *P* |
| --- | --- | --- | --- | --- | --- |
| Constant | 1004.231 | 208.427 | - | 4.818 | <0.001 |
| Thirst severity score | -8.328 | 3.367 | -0.148 | -2.473 | 0.015 |
| Pain score | -13.680 | 5.081 | -0.168 | -2.692 | 0.008 |
| Mean light intensity | -1.461 | 0.270 | -0.402 | -5.409 | <0.001 |
| Use of sleep AIDS | 66.174 | 19.248 | 0.162 | 3.438 | 0.001 |
| Noninvasive operation (times) | -7.051 | 2.074 | -0.161 | -3.400 | 0.001 |

Note: *R^2^* =0.819; Adjusted *R^2^* =0.784; *F*=23.781; *P*<0.05; - indicated that no such data existed.

Supplementary Table 4 Multivariate regression analysis of influencing factors of subjective sleep quality on Day 1

|  | Regression coefficient | Standard error | Standardized regression coefficient | *t* value | *P* |
| --- | --- | --- | --- | --- | --- |
| Constant | 149.598 | 42.325 | - | 3.535 | 0.001 |
| Pain score | -2.762 | 1.063 | -0.239 | -2.599 | 0.011 |
| Mean light intensity | -0.204 | 0.057 | -0.394 | -3.602 | <0.001 |

Note: *R^2^* =0.596; Adjusted *R^2^* =0.531; *F*=9.227; *P*<0.05; - indicated that no such data existed.

Supplementary Table 5 Correlation analysis of influencing factors of sleep quality on day 3 (*r* value)

| Items | Total sleep duration | RCSQ score |
| --- | --- | --- |
| Age | -0.006 | -0.058 |
| Gender | 0.134 | 0.049 |
| Level of education | 0.033 | 0.045 |
| Marital status | 0.022 | -0.119 |
| Working condition | 0.018 | -0.133 |
| Payment method | -0.052 | -0.116 |
| Sleep status before admission | 0.141 | -0.036 |
| Have chronic diseases | 0.039 | 0.003 |
| Have history of injury | -0.135 | 0.047 |
| APACHE-II score | -0.048 | -0.039 |
| ISS score | -0.122 | -0.098 |
| Trauma type | 0.056 | -0.008 |
| Head | 0.009 | 0.088 |
| Thorax | 0.074 | -0.007 |
| Belly | 0.211^*^ | 0.098 |
| Pelvis | 0.023 | 0.092 |
| Limbs | -0.095 | -0.078 |
| Spine | -0.020 | 0.067 |
| No operation | 0.013 | -0.118 |
| Anesthesia method | -0.001 | -0.120 |
| Body temperature | -0.422^**^ | -0.264^**^ |
| Gastrointestinal symptom | -0.207^*^ | -0.231^**^ |
| Oxygen therapy type | 0.056 | 0.053 |
| Disturbance in respiration (or dyspnea) | -0.142 | -0.139 |
| VAS score (dyspnea score) | -0.151 | -0.128 |
| Cough | -0.159 | -0.191^*^ |
| Nocturnal cough symptom score | -0.267^**^ | -0.269^**^ |
| Thirst severity score | -0.441^**^ | -0.337^**^ |
| Pain score | -0.664^**^ | -0.424^**^ |
| Self-care Score (Barthel) | 0.109 | 0.050 |
| Myodynamia | -0.138 | -0.064 |
| Restrained condition | 0.167^*^ | 0.030 |
| Catheter slip score | 0.058 | -0.084 |
| Minimum noise value | 0.059 | 0.128 |
| Maximum noise value | -0.182^*^ | -0.263^**^ |
| Mean noise value | -0.702^**^ | -0.543^**^ |
| Minimum ward illumination | 0.028 | -0.022 |
| Maximum ward illumination | 0.045 | -0.061 |
| Mean light intensity value | -0.693^**^ | -0.443^**^ |
| Minimum ward temperature value | -0.178^*^ | -0.135 |
| Maximum ward temperature value | -0.200^*^ | -0.178^*^ |
| Mean ward temperature value | -0.215^*^ | -0.200^*^ |
| Minimum ward humidity | -0.008 | 0.052 |
| Maximum ward humidity | 0.117 | 0.105 |
| Mean ward humidity | 0.064 | 0.031 |
| Ward type | -0.227^**^ | -0.206^*^ |
| Whether entertainment facilities are available in the room | -0.204^*^ | -0.221^**^ |
| Hospital anxiety scale score | -0.461^**^ | -0.300^**^ |
| Hospital depression scale score | -0.344^**^ | -0.279^**^ |
| Day visiting form | 0.296^**^ | 0.095 |
| Visiting duration | 0.453^**^ | 0.286^**^ |
| Use of vasoactive drugs | 0.014 | 0.090 |
| Pain medication use | -0.028 | -0.060 |
| Use of sleep AIDS | 0.378^**^ | 0.249^**^ |
| Invasive operation | -0.123 | -0.195^*^ |
| Noninvasive operation | -0.198^*^ | -0.096 |

Note: ^*^*P*<0.05, ^**^*P*<0.01. RCSQ: Richards-Campbell sleep questionnaire; APACHE-II: Acute Physiology and Chronic Health Evaluation II; ISS: injury severity score; VAS: visual analogue scale.

Supplementary Table 6 Multiple regression analysis of factors influencing objective sleep quality on day 3

|  | Regression coefficient | Standard error | Standardized regression coefficient | *t* value | *P* |
| --- | --- | --- | --- | --- | --- |
| Constant | 1135.002 | 241.104 | - | 4.708 | <0.001 |
| Thirst severity score | -7.589 | 3.096 | -0.14 | -2.451 | 0.016 |
| Pain score | -17.857 | 4.208 | -0.256 | -4.243 | <0.001 |
| Mean noise value | -8.895 | 3.145 | -0.193 | -2.828 | 0.006 |
| Mean light intensity value | -0.883 | 0.228 | -0.259 | -3.867 | <0.001 |
| Use of sleep AIDS | 23.312 | 6.972 | 0.167 | 3.343 | 0.001 |

Note: *R^2^* =0.759; Adjusted *R^2^* =0.716; *F*=17.529; *P*<0.05; - indicated that no such data existed.

Supplementary Table 7 Multivariate regression analysis of influencing factors of subjective sleep quality on day 3

|  | Regression coefficient | Standard error | Standardized regression coefficient | *t* value | *P* |
| --- | --- | --- | --- | --- | --- |
| Constant | 275.691 | 57.11 | - | 4.827 | <0.001 |
| Maximum noise value | -0.957 | 0.475 | -0.151 | -2.017 | 0.046 |
| Mean noise value | -2.122 | 0.78 | -0.286 | -2.72 | 0.007 |

Note: *R^2^* =0.424; Adjusted *R^2^* =0.337; *F*=4.904; *P*<0.05; - indicated that no such data existed.

Supplementary Table 8 Correlation analysis of influencing factors of sleep quality on day 5 (*r* value)

| Items | Total sleep duration | RCSQ score |
| --- | --- | --- |
| Age | -0.113 | -0.094 |
| Gender | -0.003 | -0.042 |
| Level of education | 0.124 | 0.105 |
| Marital status | -0.058 | -0.122 |
| Working condition | 0.010 | -0.018 |
| Payment method | 0.447^**^ | 0.193^*^ |
| Sleep status before admission | -0.090 | -0.231^**^ |
| Have chronic diseases | -0.042 | -0.028 |
| Have history of injury | -0.225^**^ | -0.125 |
| APACHE-II score | 0.108 | 0.128 |
| ISS score | -0.441^**^ | -0.251^**^ |
| Trauma type | 0.043 | 0.024 |
| Head | 0.130 | -0.047 |
| Thorax | -0.009 | -0.025 |
| Belly | 0.088 | -0.039 |
| Pelvis | -0.054 | -0.056 |
| Limbs | -0.466^**^ | -0.428^**^ |
| Spine | 0.064 | 0.102 |
| No operation | -0.012 | -0.009 |
| Anesthesia method | -0.030 | -0.064 |
| Body temperature | -0.264^**^ | -0.066 |
| Gastrointestinal symptom | -0.218^**^ | -0.062 |
| Oxygen therapy type | 0.265^**^ | 0.125 |
| Disturbance in respiration (or dyspnea) | -0.305^**^ | -0.171^*^ |
| VAS score (dyspnea score) | -0.348^**^ | -0.209^*^ |
| Cough | -0.149 | -0.061 |
| Nocturnal cough symptom score | -0.255^**^ | -0.097 |
| Thirst severity score | -0.331^**^ | -0.266^**^ |
| Pain score | -0.577^**^ | -0.358^**^ |
| Self-care Score (Barthel) | 0.002 | -0.021 |
| Myodynamia | -0.143 | -0.004 |
| Restrained condition | 0.054 | -0.063 |
| Catheter slip score | -0.091 | -0.062 |
| Minimum noise value | 0.016 | 0.014 |
| Maximum noise value | -0.050 | -0.095 |
| Mean noise value | -0.561^**^ | -0.363^**^ |
| Minimum ward illumination | -0.140 | -0.155 |
| Maximum ward illumination | 0.073 | -0.052 |
| Mean light intensity value | -0.565^**^ | -0.223^**^ |
| Minimum ward temperature value | -0.128 | -0.051 |
| Maximum ward temperature value | -0.113 | -0.040 |
| Mean ward temperature value | -0.185^*^ | -0.081 |
| Minimum ward humidity | 0.007 | -0.012 |
| Maximum ward humidity | 0.060 | 0.054 |
| Mean ward humidity | 0.036 | 0.044 |
| Ward type | -0.139 | -0.143 |
| Whether entertainment facilities are available in the room | -0.127 | -0.125 |
| Hospital anxiety scale score | 0.094 | -0.013 |
| Hospital depression scale score | -0.226^**^ | -0.127 |
| Day visiting form | 0.218^**^ | 0.203^*^ |
| Visiting duration | 0.378^**^ | 0.257^**^ |
| Use of vasoactive drugs | 0.004 | -0.067 |
| Pain medication use | 0.152 | 0.072 |
| Use of sleep AIDS | 0.368^**^ | 0.241^**^ |
| Invasive operation | -0.226^**^ | -0.111 |
| Noninvasive operation | -0.272^**^ | -0.033 |

Note: ^*^*P*<0.05, ^**^*P*<0.01. RCSQ: Richards-Campbell sleep questionnaire; APACHE-II: Acute Physiology and Chronic Health Evaluation II; ISS: injury severity score; VAS: visual analogue scale.

Supplementary Table 9 Multiple regression analysis of factors influencing objective sleep quality on day 5

|  | Regression coefficient | Standard error | Standardized regression coefficient | *t* value | *P* |
| --- | --- | --- | --- | --- | --- |
| Constant | 1342.945 | 209.144 | - | 6.421 | <0.001 |
| Payment method | 15.68 | 4.978 | 0.187 | 3.15 | 0.002 |
| ISS score | -1.154 | 0.578 | -0.126 | -1.995 | 0.048 |
| Trauma site (extremities) | -23.102 | 8.976 | -0.156 | -2.574 | 0.011 |
| Mean noise value | -11.441 | 2.926 | -0.261 | -3.91 | <0.001 |
| Average illuminance value | -0.687 | 0.233 | -0.19 | -2.955 | 0.004 |
| Mean ward temperature | -10.095 | 4.739 | -0.122 | -2.13 | 0.035 |
| Use of sleep AIDS | 15.983 | 4.723 | 0.193 | 3.384 | 0.001 |

Note: *R^2^* =0.719; Adjusted *R^2^* =0.668; *F*=14.252; *P*<0.05; - indicated that no such data existed. ISS: injury severity score.

Supplementary Table 10 Multivariate regression analysis of influencing factors of subjective sleep quality on day 5

|  | Regression coefficient | Standard error | Standardized regression coefficient | *t* value | *P* |
| --- | --- | --- | --- | --- | --- |
| Constant | 150.994 | 40.851 | - | 3.696 | <0.001 |
| Sleep status before admission | -11.201 | 3.327 | -0.251 | -3.367 | 0.001 |
| Trauma site (extremities) | -8.511 | 2.236 | -0.318 | -3.806 | <0.001 |
| Mean noise value | -1.463 | 0.694 | -0.184 | -2.108 | 0.037 |
| Use of sleep AIDS | 2.694 | 1.143 | 0.179 | 2.358 | 0.020 |

Note: *R^2^* =0.388; Adjusted *R^2^* =0.324; *F*=6.093; *P*<0.05; - indicated that no such data existed.

Supplementary Table 11 Correlation analysis of influencing factors of sleep quality on day 7 (*r* value)

| Items | Total sleep duration | RCSQ score |
| --- | --- | --- |
| Age | -0.095 | -0.069 |
| Gender | -0.038 | 0.018 |
| Level of education | 0.088 | 0.062 |
| Marital status | 0.032 | -0.032 |
| Working condition | -0.022 | -0.054 |
| Payment method | 0.212^*^ | 0.175^*^ |
| Sleep status before admission | -0.013 | -0.229^**^ |
| Have chronic diseases | -0.198^*^ | -0.170^*^ |
| Have history of injury | -0.084 | -0.109 |
| APACHE-II score | -0.003 | 0.069 |
| ISS score | -0.170^*^ | -0.166 |
| Trauma type | 0.087 | 0.076 |
| Head | 0.061 | -0.050 |
| Thorax | -0.129 | -0.140 |
| Belly | 0.077 | -0.053 |
| Pelvis | 0.052 | 0.011 |
| Limbs | -0.200^*^ | -0.311^**^ |
| Spine | 0.153 | 0.074 |
| No operation | 0.005 | 0.018 |
| Anesthesia method | -0.036 | 0.002 |
| Body temperature | 0.048 | 0.051 |
| Gastrointestinal symptom | -0.182^*^ | -0.171^*^ |
| Oxygen therapy type | 0.059 | 0.079 |
| Disturbance in respiration (or dyspnea) | -0.048 | -0.121 |
| VAS score (dyspnea score) | 0.044 | 0.011 |
| Cough | -0.059 | 0.001 |
| Nocturnal cough symptom score | 0.080 | 0.118 |
| Thirst severity score | -0.144 | -0.067 |
| Pain score | -0.421^**^ | -0.292^**^ |
| Self-care Score (Barthel) | -0.042 | 0.033 |
| Myodynamia | -0.016 | 0.017 |
| Restrained condition | -0.014 | -0.093 |
| Catheter slip score | 0.003 | -0.069 |
| Minimum noise value | -0.034 | 0.080 |
| Maximum noise value | 0.050 | -0.108 |
| Mean noise value | -0.486^**^ | -0.590^**^ |
| Minimum ward illumination | -0.139 | -0.229^**^ |
| Maximum ward illumination | -0.051 | -0.110 |
| Mean light intensity value | -0.030 | -0.079 |
| Minimum ward temperature value | 0.088 | 0.165 |
| Maximum ward temperature value | 0.148 | 0.136 |
| Mean ward temperature value | -0.074 | -0.083 |
| Minimum ward humidity | 0.027 | -0.034 |
| Maximum ward humidity | 0.024 | -0.008 |
| Mean ward humidity | 0.100 | 0.082 |
| Ward type | -0.015 | -0.120 |
| Whether entertainment facilities are available in the room | 0.079 | 0.087 |
| Hospital anxiety scale score | -0.474^**^ | -0.298^**^ |
| Hospital depression scale score | -0.140 | 0.018 |
| Day visiting form | 0.072 | 0.038 |
| Visiting duration | -0.276^**^ | -0.395^**^ |
| Use of vasoactive drugs | -0.006 | -0.025 |
| Pain medication use | 0.071 | 0.094 |
| Use of sleep AIDS | 0.656^**^ | 0.317^**^ |
| Invasive operation | -0.079 | -0.112 |
| Noninvasive operation | -0.073 | -0.017 |

Note: ^*^*P*<0.05, ^**^*P*<0.01. RCSQ: Richards-Campbell sleep questionnaire; APACHE-II: Acute Physiology and Chronic Health Evaluation II; ISS: injury severity score; VAS: visual analogue scale.

Supplementary Table 12 Multiple regression analysis of influencing factors of objective sleep quality on day 7

|  | Regression coefficient | Standard error | Standardized regression coefficient | *t* value | *P* |
| --- | --- | --- | --- | --- | --- |
| Constant | 708.894 | 93.864 | - | 7.552 | <0.001 |
| Pain score | -7.662 | 2.965 | -0.143 | -2.584 | 0.011 |
| Mean noise value | -6.311 | 1.658 | -0.223 | -3.806 | <0.001 |
| Hospital anxiety scale score | -5.172 | 1.387 | -0.206 | -3.728 | <0.001 |
| Use of sleep AIDS | 37.232 | 3.951 | 0.531 | 9.423 | <0.001 |

Note: *R^2^* =0.675; Adjusted *R^2^* =0.649; *F*=26.571; *P*<0.05; - indicated that no such data existed.

Supplementary Table 13 Multivariate regression analysis of influencing factors of subjective sleep quality on day 7

|  |  | Regression coefficient | Standard error | Standardized regression coefficient | *P* value |
| --- | --- | --- | --- | --- | --- |
| Constant | 193.998 | 23.568 | - | 8.232 | <0.001 |
| Sleep status before admission | -6.2 | 2.559 | -0.16 | -2.423 | 0.017 |
| Trauma site (extremities) | -3.358 | 1.644 | -0.145 | -2.043 | 0.043 |
| Mean noise value | -2.162 | 0.419 | -0.377 | -5.158 | <0.001 |
| Visiting duration | -3.382 | 1.286 | -0.177 | -2.629 | 0.010 |
| Use of sleep AIDS | 2.816 | 0.994 | 0.198 | 2.835 | 0.005 |

Note: *R^2^* =0.509; Adjusted *R^2^* =0.467; *F*=11.979; *P*<0.05; - indicated that no such data existed.
